# The evolutionary dynamics and epidemiological history of hepatitis C virus genotype 6, including unique strains from the Li community of Hainan Island, China

**DOI:** 10.1093/ve/veac012

**Published:** 2022-02-16

**Authors:** Ru Xu, Elihu Aranday-Cortes, E Carol McWilliam Leitch, Joseph Hughes, Joshua B Singer, Vattipally Sreenu, Lily Tong, Ana da Silva Filipe, Connor G G Bamford, Xia Rong, Jieting Huang, Min Wang, Yongshui Fu, John McLauchlan

**Affiliations:** MRC-University of Glasgow Centre for Virus Research, Sir Michael Stoker Building, Garscube Campus, 464 Bearsden Road, Glasgow G61 1QH, UK; MRC-University of Glasgow Centre for Virus Research, Sir Michael Stoker Building, Garscube Campus, 464 Bearsden Road, Glasgow G61 1QH, UK; MRC-University of Glasgow Centre for Virus Research, Sir Michael Stoker Building, Garscube Campus, 464 Bearsden Road, Glasgow G61 1QH, UK; MRC-University of Glasgow Centre for Virus Research, Sir Michael Stoker Building, Garscube Campus, 464 Bearsden Road, Glasgow G61 1QH, UK; MRC-University of Glasgow Centre for Virus Research, Sir Michael Stoker Building, Garscube Campus, 464 Bearsden Road, Glasgow G61 1QH, UK; MRC-University of Glasgow Centre for Virus Research, Sir Michael Stoker Building, Garscube Campus, 464 Bearsden Road, Glasgow G61 1QH, UK; MRC-University of Glasgow Centre for Virus Research, Sir Michael Stoker Building, Garscube Campus, 464 Bearsden Road, Glasgow G61 1QH, UK; MRC-University of Glasgow Centre for Virus Research, Sir Michael Stoker Building, Garscube Campus, 464 Bearsden Road, Glasgow G61 1QH, UK; Guangzhou Blood Center, Institute of Clinical Blood Transfusion, Guangzhou Blood Center, 31 LuYuan Road, Guangzhou, Guangdong 510095, P.R. China; Guangzhou Blood Center, Institute of Clinical Blood Transfusion, Guangzhou Blood Center, 31 LuYuan Road, Guangzhou, Guangdong 510095, P.R. China; Guangzhou Blood Center, Institute of Clinical Blood Transfusion, Guangzhou Blood Center, 31 LuYuan Road, Guangzhou, Guangdong 510095, P.R. China; Guangzhou Blood Center, Institute of Clinical Blood Transfusion, Guangzhou Blood Center, 31 LuYuan Road, Guangzhou, Guangdong 510095, P.R. China; MRC-University of Glasgow Centre for Virus Research, Sir Michael Stoker Building, Garscube Campus, 464 Bearsden Road, Glasgow G61 1QH, UK; Guangzhou Blood Center, Institute of Clinical Blood Transfusion, Guangzhou Blood Center, 31 LuYuan Road, Guangzhou, Guangdong 510095, P.R. China

**Keywords:** hepatitis C virus, HCV, genotype 6, evolution, Hainan Island, phylogenetics, Bayesian inference

## Abstract

Hepatitis C virus (HCV) is a highly diverse pathogen that frequently establishes a chronic long-term infection, but the origins and drivers of HCV diversity in the human population remain unclear. Previously unidentified strains of HCV genotype 6 (gt6) were recently discovered in chronically infected individuals of the Li ethnic group living in Baisha County, Hainan Island, China. The Li community, who were early settlers on Hainan Island, has a distinct host genetic background and cultural identity compared to other ethnic groups on the island and mainland China. In this report, we generated 33 whole virus genome sequences to conduct a comprehensive molecular epidemiological analysis of these novel gt6 strains in the context of gt6 isolates present in Southeast Asia. With the exception of one gt6a isolate, the Li gt6 sequences formed three novel clades from two lineages which constituted 3 newly assigned gt6 subtypes and 30 unassigned strains. Using Bayesian inference methods, we dated the most recent common ancestor for all available gt6 whole virus genome sequences to approximately 2767 bce (95 per cent highest posterior density (HPD) intervals, 3670–1397 bce), which is far earlier than previous estimates. The substitution rate was 1.20 × 10^−4^ substitutions/site/year (s/s/y), and this rate varied across the genome regions, from 1.02 × 10^−5^ s/s/y in the 5’untranslated region (UTR) region to 3.07 × 10^−4^ s/s/y in E2. Thus, our study on an isolated ethnic minority group within a small geographical area of Hainan Island has substantially increased the known diversity of HCV gt6, already acknowledged as the most diverse HCV genotype. The extant HCV gt6 sequences from this study were probably transmitted to the Li through at least three independent events dating perhaps from around 4,000 years ago. This analysis describes deeper insight into basic aspects of HCV gt6 molecular evolution including the extensive diversity of gt6 sequences in the isolated Li ethnic group.

## 1. Introduction

Hepatitis C virus (HCV) causes a chronic infection in an estimated 71 million individuals across the globe (HCV Polaris Observatory 2017). Annually, there are approximately 1.75 million new HCV infections and 400,000 deaths associated with chronic infection, most of which arise from the development of life-threatening complications such as cirrhosis and hepatocellular carcinoma (Lauer and Walker 2001). Thus, chronic HCV infection exerts a high disease burden in the human population.

HCV is a single-stranded, positive-sense RNA virus with a genome of approximately 9,600 nucleotides in length. A combination of the high error rate of the virus-encoded RNA-dependent RNA polymerase, host selection pressures, occasional recombination, and population genetic factors such as gene flow and founder effects have driven extensive diversity of the virus. HCV has been classified into 8 genotypes (gt1–gt8) (Borgia et al. 2018) and further divided into 90 subtypes (Smith et al. 2014) (https://talk.ictvonline.org/ictv_wikis/flaviviridae/w/sg_flavi/634/table-1---confirmed-hcv-genotypes-subtypes-may-2019). Gt6 is the most diverse genotype with 31 assigned subtypes (gt6a–gt6xh) and 19 unassigned strains that do not yet meet the requirements for new subtype designation. With the exception of Malaysia, HCV gt6 is endemic in the Indochinese Peninsula (Myanmar, Laos, Cambodia, Thailand, and Vietnam) and accounts for 35–95 per cent of HCV infections based on estimates for each country (HCV Polaris Observatory 2017). The extensive diversity of subtypes found in Southeast Asia suggests that gt6 may originate from this region (Thong et al. 2014). A comprehensive study using partial genome sequences revealed that in its evolutionary history, gt6 has displayed both substantial phylogeographic structure and a biphasic epidemiology dating before and during the 20th century (Pybus et al. 2009).

In previous surveys of HCV sequences found on Hainan Island, a Chinese province that lies in the South China Sea, diverse, novel gt6 strains were identified with a high prevalence among elderly members of the Li ethnic minority group of Baisha County (An et al. 2014; Wu et al. 2018; Xu et al. 2019). Based on phylogenetic data from partial sequences, the extant Li sequences clustered with only a few sequences from other geographical regions, and it was estimated that these lineages coalesced 600–900 years ago (An et al. 2014). The Li ethnic group (also known as the Hlai) represents approximately 15 per cent of the total population of Hainan Island and mainly occupies the central and southern regions of the island. They are thought to descend from the earliest settlers on Hainan, which likely occurred from 5000 to 22000 bce (Li et al. 2008; Peng et al. 2011). The Li group can be further subdivided into five subgroups (Ha, Gei, Zwn, Moifau, and Jiamao) and have distinct geographical distributions in the regions that they occupy on the island (Li et al. 2008). From host genetic studies, these five subgroups are very closely inter-related and have haplotype profiles similar to historical populations from southern China (Li et al. 2008; Peng et al. 2011; Chen et al. 2017). However, their genetic profiles are distinct from the Chinese Han who mainly occupy the northern region of Hainan. Thus, the Li group is one of the indigenous groups who settled on Hainan and have been largely isolated over extensive periods of time due to the terrain on the island as well as cultural and linguistic barriers.

In this study, we generated 33 HCV gt6 whole virus genome sequences from samples obtained from members of the Li community. By combining them with gt6 whole-genome sequences from public databases, we examined the phylogeny of the Li HCV subtypes contextually and investigated site-specific selection along the genome. Furthermore, we investigated the evolutionary dynamics for the whole gt6 genome and individual genome regions.

## 2. Results

### 2.1 HCV consensus sequence data for the Li community in Baisha County

**Figure 1.**
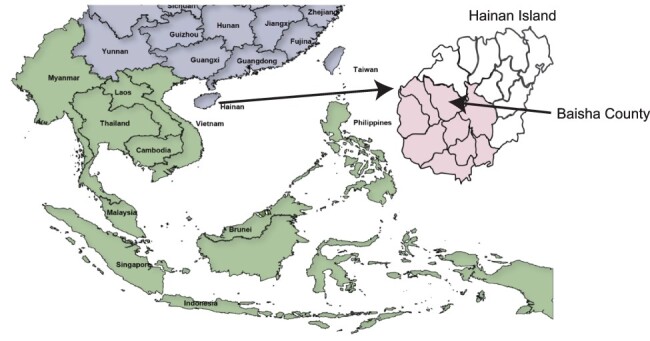
Map of the geographical location of Hainan Island and Baisha County. China and surrounding Asian countries are shown in purple and green, respectively. The expanded view of Hainan Island shows the different districts of Hainan Island with the location of Baisha County arrowed. The Li ethnic group on Hainan Island is predominantly located in the districts that are colored pink.

Previously, we described a survey of 1,682 volunteers from Baisha County on Hainan Island, China (Fig. 1), which revealed that 67 individuals (4 per cent) were chronically infected with HCV (Xu et al. 2019). Of the 46 samples available for high-throughput sequencing (HTS), all samples were collected in 2014 or 2015 from treatment-naïve patients whose average age was 75.7 ± 9.8 (Supplementary Table S1). After processing and bioinformatics assembly of the viral sequence data, 33 genomes had complete open reading frames (Supplementary Table S2). All sequences mapped to HCV gt6, and there was no evidence of recombination in the sequence data from analysis with either recombination detection program v4 (RDP4) or genetic algorithm for recombination detection (GARD) software (data not shown).

### 2.2 Sequence datasets

HCV whole virus genome reference sequences (*n* = 223) were obtained from the International Committee on Taxonomy of Viruses (ICTV) website (https://talk.ictvonline.org/), 142 of which were non-gt6 sequences. The 81 gt6 sequences were supplemented with further 57 whole virus genome gt6 sequences downloaded from international databases, and the total 138 gt6 sequences are outlined in Supplementary Table S3. Of these sequences, eight were from Li individuals from Baisha County (indicated as HN in Supplementary Table S3) (An et al. 2014; Wu et al. 2018). For presentation purposes, all sequences from the current study (*n* = 33) and previous reports from Baisha County (*n* = 8) will be referred to as dataset gt6_Bai_ (*n* = 41) and those from the other gt6 reference sequences (*n* = 130) will be denoted as dataset gt6_Ref_. In some appropriate analyses, an additional 13 gt6 sequences from Baisha County with incomplete (≤9,000 bp, highlighted in Supplementary Table S2) whole virus genomes were included. E1, NS5A, and NS5B gt6 sequences from public databases were used for maximum-likelihood (ML) inference alongside the appropriate genome region from the whole-genome gt6 datasets to give a total of 641, 373, and 351 sequences, respectively.

### 2.3 Genetic diversity in the sequences from Baisha County relative to other gt6 sequences

**Figure 2.**
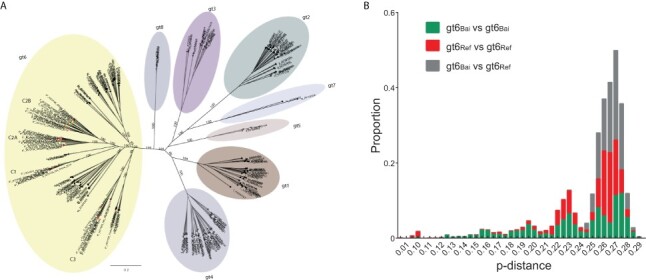
Diversity of HCV gt6_Bai_ sequences compared to non-Baisha gt6 sequences. (A) Phylogenetic tree of all HCV reference sequences and gt6_Bai_ sequences. The HCV genotypes (gt1–gt8) are highlighted with different colors. Sequences labeled with red and green solid circles represent whole virus genome (*n* = 33) and incomplete (*n* = 13) gt6_Bai_ sequences, respectively, from this study. Bootstrap support values are only shown for the major clades. (B) Comparison of the p-distance between HCV gt6_Ref_ and gt6_Bai_ sequences using SSE.

A ML phylogenetic tree (Fig. 2A) was inferred for the non-gt6 reference HCV sequences, the gt6_ref_ dataset, the gt6_Bai_ dataset, and the incomplete gt6 sequences, a total of 269 HCV sequences, 54 of which were derived from Baisha County. The gt6_Bai_ isolates were very diverse and largely distinct from previous gt6 subtypes except for one strain which clustered with gt6a sequences (6*_HCV128_Yacha) (Fig. 2A). With the exception of the gt6a isolate, the gt6_Bai_ sequences formed three distinct clades (denoted C1, C2, and C3), with C2 bifurcating into sub-clades C2A and C2B. These three clades were also apparent in phylogenetic analyses of individual genes (HCV gt6 E1 (*n* = 641), NS5A (*n* = 373), and NS5B (*n* = 351); Supplementary Figs S1–S3), which included a much larger sampling of sequences from mainland China, although the bootstrap support at deeper nodes was not as high as in the whole-genome tree, possibly due to shorter sequence lengths.

We further used the 130 whole virus genome gt6_Ref_ dataset and the 41 gt6_Bai_ dataset to construct a more detailed ML phylogenetic tree for gt6 alone, which illustrated the extent of diversity of the gt6_Bai_ sequences (Supplementary Fig. S4). Excluding the gt6a isolate (HCV128_Yacha), the other 40 gt6_Bai_ sequences occurred only in Clades C1, C2, and C3 and only 3/43 sequences in these three clades were not from the gt6_Bai_ dataset.

Clade C1 comprised seven Baisha isolates with a p-distance of between 20 and 24 per cent. Three isolates (HCV141, HCV99, and HCV79) clustered with the sequence MG879000, which belongs to the newly proposed gt6xh subtype (Wu et al. 2018), with a pairwise p-distance range between 13.2 and 15.6 per cent. The distance between two other isolates (HCV073 and HCV084) in Clade C1 was only 5.5 per cent, suggestive of a new gt6 subtype, however this would require a third isolate. Clade C2 contained 27 isolates, was more diverse than C1, and, as described above, could be divided into subclades C2A (*n* = 12) and C2B (*n* = 15). In subclade C2A, the pairwise distance range between the sequences was 12.3–22.4 per cent. The pairwise distances between two groups of isolates within clade C2A (HCV101, HCV147, and HCV153 (p-distance 12.3–13.8 per cent) and HCV92, HCV74, and HCV108 (p-distance 12.9–14.6 per cent)) met the taxonomic criteria for classifying subtypes and were provisionally designated gt6xi and gt6xj, respectively. Clade C2B included two gt6g sequences from Indonesia and Hong Kong (D63822 and DQ314806, respectively (Tokita et al. 1996; Li et al. 2006)). The three most closely related strains in this clade were HCV094, HCV139, and HCV156, which had a p-distance of 7.6–15.7 per cent, marginally outside the criteria for assigning a new subtype. Clade C3 consisted of nine sequences but did not contain any three sequences with p-distances of <15 per cent. Clade C3 contained seven sequences identified in this report, one sequence that had been characterized previously from an individual from Baisha County (MG878999 (Wu et al. 2018)) and the remaining sequence (KC844040) originated from a patient attending clinic in Guangzhou (Xu et al. 2013), the major city of Guangdong Province which is closest geographically to Hainan Island. Overall, the sequences derived from HCV-infected individuals in the Li ethnic group residing in Baisha County generated 3 novel gt6 subtypes and further 30 unassigned strains in three clades.

To compare the diversity within the gt6 sequences from Baisha County with the diversity of gt6 sequences found worldwide (predominantly from other regions in Southeast Asia), the distribution of pairwise nucleotide p-distances was calculated within and between the gt6_Bai_ and gt6_Ref_ datasets (Fig. 2B). The median p-distance (Q1, Q3) of gt6_Bai_ vs. gt6_Bai_, gt6_Ref_ vs. gt6_Ref_, and gt6_Ref_ vs. gt6_Bai_ were 0.249 (0.214, 0.268), 0.258 (0.229, 0.264), and 0.264 (0.256, 0.270), respectively, indicating similar genetic diversity between the datasets. Thus, the diversity between the gt6_Bai_ sequences was similar to that between gt6 sequences found in Southeast Asia and elsewhere across the world.

### 2.4 BEAST analysis of gt6 whole-genome sequences

#### 2.4.1 Bayesian MCMC inference of Gt6 sequences

**Figure 3.**
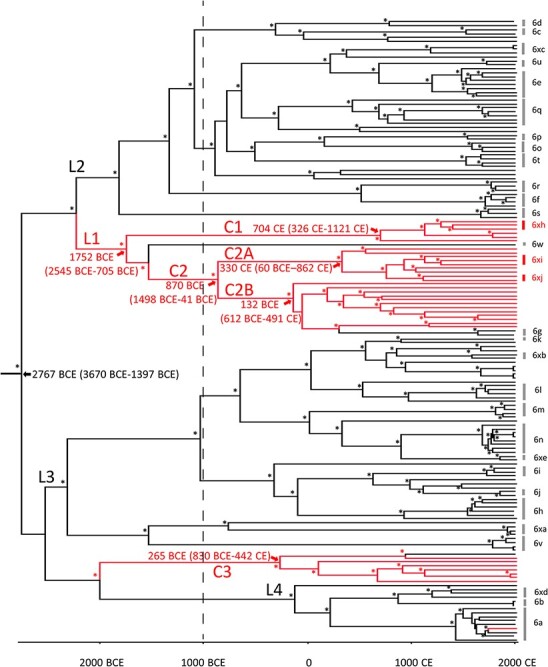
Bayesian phylogenetic tree of whole virus genome HCV gt6_Ref_ and gt6_Bai_ sequences. Sequences labeled with back and red lines represent Gt6_Ref_ and gt6_Bai_ sequences, respectively. The timescale runs from 2767 bce to 2015 ce. Predicted dates for the MRCAs for gt6, Lineage L1, and gt6_Bai_ Clades C1, C2, C2A, C2B, and C3 are indicated on the figure along with their respective 95 per cent HPD intervals. Non-Baisha lineages (L2, L3, and L4) that were used for Bayesian plot analysis shown in Supplementary Fig. S6 are indicated. Posterior probability values of >90 per cent are indicated by asterisks at the nodes.

Phylostems (Doizy et al. 2020) was used to investigate temporal signaling in an ML tree of the gt6_Ref_ and gt6_Bai_ whole virus genome datasets. The sequences displayed a weak statistically significant (*P* = 0.0047) linear regression of root-to-tip (RtT) genetic distance against sampling time with a positive slope, suggesting that the sequences were evolving in a time-correlated manner (Supplementary Fig. S5). The slope of the line suggested an evolutionary rate of 1.5 × 10^−3^ s/s/y. Extensive scatter from the regression line suggested a relaxed molecular clock would be the most appropriate model to use for Bayesian Markov Chain Monte Carlo (MCMC) inference. We therefore inferred a maximum clade credibility phylogeny of HCV gt6 whole virus genome sequences using Bayesian analysis in the Bayesian Evolutionary Analysis by Sampling Trees (BEAST) program platform (Fig. 3). The analyzed sequences comprised the gt6_Ref_ and gt6_Bai_ datasets but with seven and two sequences excluded respectively due to sequence gaps which affected the analysis during trials (Supplementary Table S3). The estimated date of the most recent common ancestor (MRCA) of gt6 was 2767 bce, with 95 per cent HPD intervals (HPD_95_) of 3670 to 1397 bce. The substitution rate of gt6 was calculated as 1.20 × 10^−4^ s/s/y (HPD_95_ 1.00–1.62 × 10^−4^) (Supplementary Table S4) using a constant population size coalescent model which is similar to the previous estimates for the core and NS5B genes of gt6 (Pybus et al. 2009); a similar value was also obtained with a Bayesian skyline coalescent model (1.19 × 10^−4^ s/s/y (HPD_95_ 1.00–1.58 × 10^−4^)). The coefficient of variation (CoV), a measure of rate heterogeneity among lineages, was 0.236 (HPD_95_, 0.204–0.27) which is consistent with rate heterogeneity among the gt6 lineages (Supplementary Table S4). A covariance value close to and spanning zero (−0.057, HPD_95_ −0.171 to 0.05) implies that the substitution rate heterogeneity is randomly distributed across the phylogeny. The Baisha sequence-rich clades C1, C2, and C3, identified in the ML phylogeny, were also apparent in this time-correlated phylogeny, with MRCAs of 704 ce (HPD_95_, 326–1121 ce), 870 bce (HPD_95_, 1498 bce to 41 ce), and 265 bce (HPD_95_, 830 bce to 442 ce), respectively. Clades C1 and C2 evolved from a common lineage (L1) with an MRCA of 1752 bce (HPD_95_ 2545 to 705 bce).

#### 2.4.2 Partition model of MCMC analysis

The gt6 sequences were partitioned into ten coding regions, and the 5’UTR region for Bayesian analysis and the results are detailed in Supplementary Table S4. The substitution rate for each region ranged from 1.02 × 10^−5^ (HPD_95_ 7.12 × 10^−6^–1.37 × 10^−5^) for the 5’UTR region to 3.07 × 10^−4^ (HPD_95_ 2.79 × 10^−4^–3.34 × 10^−4^) for the E2 region. The rank of the median rates was 5’UTR < core < NS5B < NS4B < NS4A < NS3 < NS5A < E1 < NS2 < p7 < E2. The CoV values for the coding regions were moderate as expected, ranging from 0.216 (NS4B) to 0.654 (NS5B); however the 5’UTR substitution rate heterogeneity among lineages was much greater (2.008). To eliminate the possibility that the elevated 5’UTR CoV value was a consequence of missing data, the Bayesian analysis was repeated twice, first excluding seven taxa lacking >50 nucleotides and secondly excluding the first 50 nucleotides from all taxa, where most of the missing data were observed. In both instances, the CoV was similar to the original value of 1.45 (1.53 and 1.38, respectively). The most likely explanation for the high CoV value is that, although the substitution rate is much lower for the 5’UTR compared to the other genome regions, the variability among lineage rates is only slightly lower (5’UTR substitution rate SD, 2.39 × 10^−5^; other regions, 3.06 × 10^−5^–1.29 × 10^−4^), as previously explained in mitochondrial phylogenetics (Duchene et al. 2011). The covariance for all regions was close to zero (range, −0.0385 in NS2 to 0.0686 in E2).

#### 2.4.3 Bayesian skyline analysis

**Figure 4.**
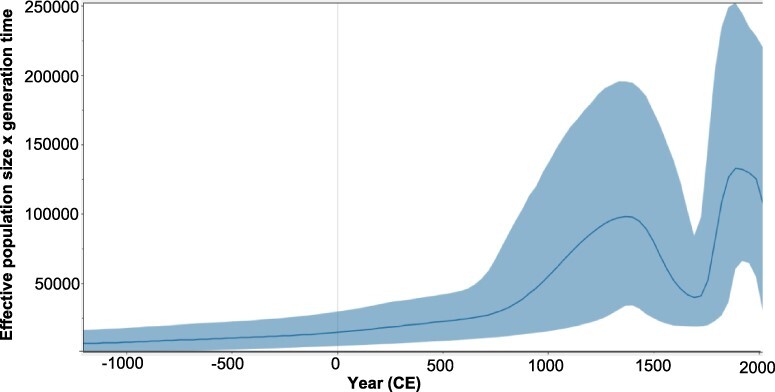
Bayesian skyline plot showing the predicted epidemic history of HCV gt6. The epidemic history was estimated from whole virus genome gt6_Ref and_ gt6_Bai_ sequences. The solid black line in the middle of the figure represents the estimated effective number of infections over time. The blue area represents the 95 per cent highest posterior density confidence intervals for this estimate.

The epidemic history of gt6 was estimated in a Bayesian skyline plot of the gt6_Bai_ and gt6_Ref_ whole-genome sequences (Fig. 4). This plot represents the effective population (*Ne*, analogous to the number of infections) through time, recorded every 32.5 years since the estimated MRCA of genotype 6. Prior to approximately 750 ce, the *Ne* increased steadily over time and then it rapidly increased, peaking in 1365 ce. Subsequently the *Ne* declined in the following 325 years to less than half the number of infections. A second sustained period of rapid expansion in *Ne* occurred shortly afterward (1723 ce onwards), peaking in 1918 ce and then decreasing until 1983 ce. Since then, the rate of decline has accelerated to the present time. To determine whether the epidemic history of the gt6_Bai_ sequences followed the same pattern as non-Baisha sequences, we compared skyline plots for three lineages (L2, L3, and L4 in Fig. 3) with Baisha Lineages L1 and Clade C3. The results showed that the epidemic histories for L2 and L3 (Supplementary Fig. S6) followed a similar pattern to that seen in Fig. 4 for the combined gt6 sequences although Lineage L4, which had a more recent MRCA than either L2 or L3 and contained fewer gt6 strains only showed a rapid rise from ∼1750 ce onward. For the Baisha sequences, there were rapid increases in population size from around 1200 and 800 ce for Lineage L1 and Clade C3, respectively (Supplementary Fig. S6). However, there was no decline in population size as observed for L2 and L3 at around 1500–1700 ce. Thus, the epidemic history for gt6 in Baisha County appears to follow a different course from gt6 strains isolated from the Indochinese Peninsula.

### 2.5 Site-specific selection of amino acid residues in the HCV polyprotein

Analysis of the 33 whole virus genome gt6_Bai_ sequences with mixed effects model of evolution (MEME) indicated purifying selection pressure at 41 amino acid sites (data not shown). Six of these sites are located within human cytotoxic T lymphocytes (CTLs) and helper T-cell (T_H_) epitopes. Among these sites, amino acid positions 10, 72, and 75 are located in the core-coding region, which is targeted by CTL restricted human leukocyte antigen (HLA) type A11, and a restricted T_H_ epitope recognized by HLA type DR Site 533 in E2 is located in a region targeted by HLA type B60; Sites 2,570 and 2,573 in NS5B are within a known epitope target for HLA type B55. The distribution of HLA class I and II alleles has previously been shown to differ between individuals of Li ethnicity and Han Chinese people, including an increased frequency of A*11:01 and B*55:02, although the latter was not found to be significant (Pan et al. 2011). Analysis of a combination of Clades C1 and C2 further identified two amino acid positions (Positions 586 and 2,023 in E2 and NS5A, respectively) under positive selection, neither of which lay in a known epitope (Table 1). Examination of sequences in Clade C3 alone failed to reveal any additional positively selected sites.

**Table 1.** Site-specific selection of amino acids in the polyprotein encoded by gt6_Bai_ isolates.

Gene	AA position	AA residue	All gt6_Bai_ clades	Clades C1 + C2
Core	10^a^	K^a^	√	√
Core	72^a^	E^a^	√	√
Core	75^a^	T^a^	√	√
E2	533^b^	D^b^	√	
E2	586	F		√
NS5A	2,023	G		√
NS5B	2,570^b^	E^b^	√	
NS5B	2,573^b^	G^b^	√	

Amino acid position and residue are based on the HCV gt1a H77 reference sequence.

a Sites that are associated with CTL epitopes.

b Sites that are in epitope targets of HCV gt1a, gt1b, and gt2a sequences.

### 2.6 The epidemiology of HCV RAS in HCV gt6_Bai_ sequences

HCV can be effectively treated using combinations of drugs called direct acting antivirals (DAAs). Although these drugs are highly efficacious, resistant viral variants occur giving rise to resistance-associated substitutions (RASs), which also can arise independently in treatment-naïve individuals. Since there are few reports of outcomes from DAA therapy for gt6, we used two sources for identifying potential RAS; firstly, the guidelines published by European Association for the Study of the Liver (EASL) (https://easl.eu/wp-content/uploads/2018/10/HepC-English-report.pdf) and secondly the HCV-GLUE online resource (Singer et al. 2019), which regularly updates\ reports on RAS from both *in vivo* and *in vitro* studies. Despite all samples originating from treatment-naïve individuals, we identified the prevalence of baseline NS3, NS5A, and NS5B RAS to DAA therapy in the gt6_Bai_ sequences from our HTS data with >15 per cent cut-off in the frequency of RAS in the intra-host viral population (Table 2). The NS3 RAS, D168E, which gives a modest increase in drug resistance *in vitro,* was identified in nine (19.6 per cent) individuals. In the context of an infectious gt2a/gt6a recombinant, this RAS had 3- and 6-fold higher half maximal inhibitory concentration (IC_50_) values with the NS3 inhibitors paritaprevir and grazoprevir, respectively (Serre et al. 2016). There were more RASs in NS5A at the >15 per cent cut-off. These occurred at Positions 28 (M/V/F), 30 (H), 31 (M/I), 58 (A/S), and 93 (S) and were present with a prevalence of between 2.2 and 34.8 per cent in the gt6_Bai_ sequences (Table 2). In 13 individuals (28 per cent), we identified two RASs in their NS5A viral sequences, which could increase possible resistance to NS5A antivirals. We also observed some specificity in the distribution of RAS in the gt6_Bai_ clades (Table 2). For example, the 168E RAS in NS3 was exclusively found in Clade C2B and some NS5A RASs were specific for Clade C2 (28M, 28F, and 58S) while the 93S RAS was only found in Clade C3. The gt6_Bai_ sequences did not contain any RAS in NS5B, the other major HCV genome region targeted by DAAs.

**Table 2.** Prevalence of RAS where the minor variant frequency is above 15 per cent in NS3 and NS5A genes in HCV gt6_Bai_ sequences (*n* = 46).

HCV gene	RAS^a^	RAS prevalence	Distribution in clades
NS3	168E	9 (19.6%)	C2B
NS5A	28M	10 (21.7%)	C2A/C2B
	28V	16 (34.8%)	C1/C2A
	28F	2 (4.4%)	C2A/C2B
	30H	1 (2.2%)	Gt6a
	31M	6 (13%)	C1
	31I	2 (4.4%)	C2B
	58A	4 (8.7%)	C2A/C3
	58S	3 (6.5%)	C2A/C2B
	93S	9 (19.6%)	C3

a RASs were identified from variants that enhanced DAA resistance for gt6 strains in both the EASL recommendations and HCV-GLUE resource.

## 3. Discussion

In this study we conducted a comprehensive investigation of the evolutionary dynamics and epidemiology of HCV gt6 using publicly available whole-genome virus sequences combined with diverse gt6 sequences obtained from the Li ethnic group of Baisha County, Hainan Island, China.

The molecular evolution of HCV gt6 has previously only been investigated using single genome regions (Smith et al. 1997) or two concatenated partial genome regions (Pybus et al. 2009). Utilizing Bayesian MCMC phylogenetic methods, we analyzed 162 whole-genome sequences, which were almost exclusively derived from individuals originating from Southeast Asia, and 39 of which were obtained from individuals of Li ethnicity. The substitution rate of the coding region of the gt6 sequences was 1.20 × 10^−4^ s/s/y, which is similar to the rate (1.8 × 10^−4^ s/s/y) previously obtained for the partial core region but less than the rate determined for NS5B (3.3 × 10^−4^ s/s/y) (Pybus et al. 2009). In the partitioned model, we determined substitution rates of 8.90 × 10^−5^ and 1.38 × 10^−4^ s/s/y for the core and NS5B regions which are similar to the values obtained by Pybus et al. (2009). In relation to other HCV genotypes, the substitution rate of whole-genome virus gt6 was less than that of gt4 sequences in Africa (1.35 × 10^−3^ s/s/y) (Iles et al. 2014). The substitution rate values obtained by Bayesian methods were lower for all genome regions compared to the RtT rate of 1.5 × 10^−3^ s/s/y. RtT regression analyses, however, provide estimations only, and our data showed a weak correlation between divergence and sampling times. Limitations of RtT include assumptions of a constant rate, and results are based on a single tree (Yuan et al. 2013). Previous comparisons between substitution rates generated from RtT and Bayesian analyses of HCV gt1a and gt1b sequences of the full coding region and the individual genome regions identified lower substitution rates using the former method of up to 3-fold (Yuan et al. 2013). Similar to previous results (Pybus et al. 2009), for both the full genome and the coding regions, posterior estimates of the CoV, a measure of rate heterogeneity among lineages, indicate a deviation from a strict clock and, as the covariance values approximate zero, parent and child branch rates are randomly distributed. The greater 5’UTR CoV posterior value indicates substantial rate heterogeneity among the lineages in this non-coding region.

By combining the available gt6 whole virus genome sequences with the increased sequence diversity introduced with the data from the Baisha community, the posterior estimated date of the MRCA of gt6 was 2767 bce which is far earlier than previous estimates of approximately 650 ce (Pybus et al. 2009). However, the confidence intervals around the MRCA remain wide (3670 bce–1397 bce) here and previously (approximately 1000 bce–1400 ce) (Pybus et al. 2009). It is important to note that the dates are calibrated on data spanning only 22 years (earliest and latest sample dates were 1994 and 2016, respectively), which makes it difficult to estimate the deeper nodes in the tree with confidence, and thus these dates should be interpreted carefully.

The combination of whole-genome sequences from our study with those from another report (Wu et al. 2018) demonstrated that the gt6_Bai_ dataset contributed considerable diversity to HCV gt6. This is remarkable given the population size in Baisha County (∼168,000) compared with that for the Indochinese Peninsula (∼244,000,000) where gt6 is thought to originate. Excluding the single gt6a isolate, the other gt6_Bai_ sequences formed three new clades with a minimum p-distance to previously sequenced strains of 14.8 per cent to gt6g and 23.2 per cent to any other reference strain. From the Bayesian inferred phylogeny and by setting an arbitrary date of 1000 bce, there are only seven other clades that give rise to the diversity of the non-Baisha sequences found across Southeast Asia. It is interesting to speculate why HCV gt6 strains from the Baisha region demonstrate such wide diversity and why they differ so substantially from strains of this genotype sequenced in other parts of Southeast Asia, particularly geographically close regions such as south China. One interpretation is that there is insufficient sampling in regions outside of Baisha, particularly other regions in Hainan Island and mainland China. This is probably true for the whole-genome analysis, which consisted of 130 non-Baisha and 41 Baisha sequences, and more comprehensive sampling would assist in determining if the high level of diversity and geographical specificity of gt6 strains in Baisha County is repeated elsewhere. Certainly, analysis of the E1, NS5A, and NS5B genome regions (Supplementary Figs S1–S3), which included far larger numbers of non-Baisha sequences, shows a similar pattern of Baisha sequences clustering into three clades and a small number of non-Baisha sequences within these clades with the exception perhaps of C2 in the E1 tree (Supplementary Fig. S1), which had five non-Baisha sequences compared to only two in the whole-genome tree. Four of these sequences formed a clade of gt6g sequences (GenBank sequences D49748, D49758, D49751, and DQ314806) while the final sequence is the sole whole-genome sequence available for gt6w (GenBank sequence DQ278892).

Alternatively, the clustering of previously unidentified gt6 isolates from Baisha County may relate to the isolation of the Li community and certain cultural practices. Several studies examining the genetic diversity of the Li ethnic group support the idea that this community is isolated. Song et al. (2019) found a low level of genetic diversity within the Li and limited gene flow with other ethnic groups; segregation has been measured to approximately 20,000 years ago (Li et al. 2008). Moreover, ritual tattooing was common in the Li community, especially among women. Such cultural practices could be responsible for persistent transmission of HCV and explain the uniqueness of the gt6_Bai_ sequences. From the BEAST phylogeny and dating of MRCAs, we propose that there have been at least three introductions of gt6 into the Li community in Baisha County. The most recent of these was gt6a, which is likely to have occurred around 1,750 ce (HPD_95_: 1,664–1,837) and is represented by a single sequence in this study. The other transmission events are more historical, potentially dating to 4,000 years ago. The most complex history is for Clades C1, C2A, and C2B, which have an MRCA dated at around 1752 bce and evolved from Lineage L1, which incorporates gt6w (Fig. 3); gt6w is represented by a single whole-genome sequence that was isolated from an individual in Guangzhou, in the southern region of China (Lu et al. 2006) and therefore in relative close proximity to Hainan Island. The L1 lineage separates into Clades C1, C2A, and C2B, with MRCAs of 704 ce, 330 ce, and 132 bce, respectively. Thus, it is possible that HCV gt6 was introduced into the Li group at around 1752 bce and then diversified into distinct clades, which were propagated in the community. Alternatively, Clades C1, C2A, and C2B could be separate introductions of the virus into the Li group. The MRCA for Clade C3 dates to around 265 bce and has emerged from a lineage distinct from L1 with an MRCA with non-Baisha sequences of 2011 bce. Since other unique gt6 sequences have been found in the Li ethnic group (Wu et al. 2018), it is very likely that there have been additional introductions of the virus into the community.

In total, ten isolates from Baisha County cluster into three new subtypes that meet the criteria for HCV subtype nomenclature described by the ICTV (Smith et al. 2014) and are tentatively designated 6xh, 6xi, and 6xj. Other isolates, which meet the requirement for new subtype based on genetic distance but have insufficient numbers of sequences, could add to the total number of gt6 subtypes. Since there are 30 unassigned strains from our study, additional sampling from Baisha County and surrounding areas on Hainan Island may reveal far richer diversity and more strains for subtype assignment. A wider survey may also identify specific subtypes that are restricted to particular geographical regions. From a similar study in Laos, HCV gt6 was shown to be highly genetically diverse with potential new subtypes of gt6 (Hubschen et al. 2011), although no dating on the MRCAs of the isolates was performed. Excluding the 13 isolates for which we could not derive whole virus genome data, Clades C1, C2, and C3 contained 43 sequences, only three of which do not originate from the Li ethnic minority group in Baisha County. Given the lack of information about these three individuals, it is possible that they were also of Li ethnicity since this ethnic group is not located exclusively on Hainan Island.

The Bayesian skyline plot (Fig. 4) shows a bimodal expansion of the number of gt6 infections, peaking in 1365 ce and 1886 ce. This is inconsistent with a previous study (Pybus et al. 2009) analyzing concatenated partial core and partial NS5B sequences, which showed two phases of epidemic history, before and during the 20th century. One reason for the discrepancy between studies may be our analysis of whole virus genome sequences rather than partial sequences. Interestingly, we found that separating the Baisha from the non-Baisha lineages, we did not observe a bimodal distribution, which was found for two non-Baisha lineages (L2 and L3 in Fig. 3 and Supplementary Fig. S6). This suggests that the epidemic history for gt6 in Baisha County is distinct from that for gt6 strains found on the Indochinese Peninsula. Our conclusion is that this reinforces the likelihood of isolation of the Li community from other ethnic groups on the mainland being a major factor preventing genetic mixing of HCV infection. Moreover, it underlines the phylogeographic restriction for the evolution of certain gt6 subtypes, which is observed in countries in East Asia (Pybus et al. 2009).

By aligning whole virus genome gt6 sequences, our data reveal a purifying selection pressure, which we speculate is due to selection at the amino acid level for maintaining the structural and functional integrity of the virus-encoded proteins (Brown et al. 2007); however, we do not exclude the possibility that RNA structure also contributes to selection. Six sites associated with the human CTL and T_H_ epitopes were found to be under positive selection in the combined gt6_Bai_ clade C1 + C2 + C3 sequences. This selectivity underlines how interaction between HCV and the cellular immune response may drive viral adaptation (von Hahn et al. 2007). Interestingly, amino acid position 2,570 in NS5B was among the sites that were positively selected. Amino acid variation at this position in NS5B is associated with interferon lambda 4 genotype and can affect viral RNA replication (Ansari et al. 2017, 2019; Chaturvedi et al. 2019; Bamford and McLauchlan 2021). Thus, not only adaptive but also innate immunity could influence amino acid selection in gt6 sequences.

It has been proposed that transmission plays a critical role in HCV viral evolution as the virus encounters a novel host immune system, which alters the selection pressure on the viral genome and its encoded proteins (Skums et al. 2018). Compared to a study from Farci et al. (2000), there are fewer positively selected sites in the gt6_Bai_ dataset. The relatively narrow host genetic background in the Li ethnic group may not be sufficient to drive greater evolution of the virus. We also found the clades emerging from Lineage L1 (Clades C1 and C2) had additional selected sites whereas there were no selected sites specific for Clade C3. This finding could reflect the low number of sequences in Clade C3 (*n* = 9 isolates). More sampling of individuals infected with strains that are related to those in C3 would allow identification of possible variants that have evolved solely in this clade. Combining additional viral sequences with host genetic information from the infected individuals could provide unique insight into the host factors, involved in either innate or adaptive immunity, that impose selective pressure on the virus.

The HCV gt6 sequences found in Baisha individuals in this study contained potential RAS in NS3 and NS5A that could reduce treatment effectiveness. Moreover, some of the RASs were detected specifically in certain clades (Table 2). All of the samples used for our analysis were collected prior to the availability of DAA treatment. There are few reports on the outcomes of DAA therapy in gt6-infected patients but 100 per cent SVR rates have been reported for both NS3/NS5A (*n* = 43 patients) and NS5A/NS5B (*n* = 41 patients) DAA combinations (Feld et al. 2015; Puoti et al. 2018). The use of DAA treatment is now more widely available in mainland China although there are no reports on treatment access on Hainan Island. Thus, the impact of the RAS detected in the gt6_Bai_ sequences is difficult to determine in the absence of additional data.

In conclusion, we investigated the evolutionary dynamics and epidemiology of HCV gt6 using all publicly available whole-genome virus sequences and 33 new sequences derived from individuals of the Li community, Hainan Island, China. The substitution rate of the coding region of the gt6 sequences was 1.20 × 10^−4^ s/s/y, similar to previous estimates, and we determined substitution rates of between 1.02 × 10^−5^ (5’UTR) and 3.07 × 10^−4^ (E2) s/s/y for the individual genome regions using a partitioned model. The estimated date of the MRCA of the gt6 whole virus genomes was 2767 bce which is earlier than previous estimates. The diversity within a single geographic area on Hainan Island with a relatively small population was found to be extensive, with the discovery of three new gt6 subtypes (6xg, 6xh, and 6xi) and the identification of 30 strains that cannot yet be assigned to a known subtype. Moreover, our findings indicate that introduction of gt6 into the Li community has occurred through at least three events and is accompanied by extensive sequence divergence over a period of possibly several thousand years.

## 4. Materials and methods

### 4.1 Ethics and sample collection

Samples from volunteers, aged 11–95 years, were collected as part of a screening program for infectious diseases in Baisha County. The study was approved by the Institutional Review Board at the Guangzhou Blood Center, and the guidelines set by this board were strictly followed. Study protocols followed the ethical guidelines set in place by the 1975 Declaration of Helsinki and were approved by the Medical Ethics Committee of Guangzhou Blood Center.

### 4.2 High-throughput sequencing

RNA was extracted from 200 µl plasma using the Agencourt RNAdvance blood kit (Beckman Coulter), eluted into 11 μl of water and then reverse transcribed using Superscript III (Invitrogen). Random hexamers and a NEB Second Strand Synthesis kit (New England BioLabs) were used to generate double-stranded DNA. For subsequent library preparation, we used the KAPA Library Prep kit (KAPA Biosystems) with index tagging. DNA was amplified for 16 cycles by polymerase chain reaction using KAPA HiFi HotStart (KAPA Biosystems) and NEBNext Multiplex Oligos (oligonucleotides) for Illumina Index Primer Sets 1 and 2 (New England BioLabs). Libraries were quantified by Qubit (ThermoFisher), sized by TapeStation (Agilent), and pooled at equimolar concentrations. For capture, pooled libraries were enriched with the NimbleGen SeqCap EZ system (Roche) and then sequenced on an Illumina platform.

### 4.3 Bioinformatics processing of HCV sequence data

Sequence reads were checked for quality using FastQC (http://www.bioinformatics.babraham.ac.uk/projects/fastqc/) and trimmed using trim_galore (https://www.bioinformatics.babraham.ac.uk/projects/trim_galore/). The threshold for the Phred quality score was set at >30 and minimum read length was set to 75 bp. Viral genomes were assembled using an in-house *de novo* assembly pipeline (https://github.com/vbsreenu/ViralAssembly). HCV sequence reads were enriched *in silico* by removing human and ribosomal RNA sequence reads (Ribopicker, http://ribopicker.sourceforge.net/); all reads were then mapped against whole-genome HCV reference sequences using snap-aligner (https://github.com/amplab/snap) and mapped reads were extracted from the sequence dataset. These reads were iteratively assembled using the SPAdes *de novo* assembler taking random subsamples with different *k*-mer sizes. Finally, all reads were mapped against the *de novo* assembly using Tanoti (http://bioinformatics.cvr.ac.uk/tanoti.php) and majority consensus sequences were generated using SAM2CONSENSUS (https://github.com/vbsreenu/Sam2Consensus) for sites with ≥5 reads per nucleotide. The final assemblies were individually inspected in Unipro UGENE (http://ugene.net/).

### 4.4 Calculation of pairwise nucleotide p-distances and phylogenetic analysis of HCV sequence data

Consensus sequences were placed within the context of previously known genotypes by phylogenetic analysis (Smith et al. 2014) (https://talk.ictvonline.org/ictv_wikis/flaviviridae/w/sg_flavi/56/hcv-classificationv8.5.19) using MEGA v7.0 software (Kumar et al. 2016) and the ML phylogeny inference method with 500 bootstrap replicates and partial deletion for missing data. ML trees for individual HCV-encoded genes E1 (*n* = 641), NS5A (*n* = 373), and NS5B (*n* = 351), for which a greater number of sequences are available from mainland China, were also estimated to determine the influence of sampling on the topology. All available HCV gt6 whole virus genome reference sequences were downloaded from the HCV-GLUE database (http://hcv.glue.cvr.ac.uk/), which curates genotyped HCV sequences mirroring the available data in National Center for Biotechnology Information (Singer et al. 2018, 2019). The dataset of available HCV whole virus genome sequences had similar representations for each subtype except for gt6a, which contained 87 reference sequences. Nine representatives from the set of 87 gt6a reference sequences were selected according to the topology of the phylogenetic tree to avoid an over-representation of subtype gt6a when calculating the intra-gt6 distances; four out of nine of these sequences were from different regions of China (Supplementary Table S3). Consequently, a total of 138 gt6 sequences were aligned using Multiple Alignment using Fast Fourier Transform (MAFFT) (Katoh et al. 2002, 2005); details of reference sequences are shown in Supplementary Table S3 and include eight previously reported whole virus genome sequences from individuals residing in Baisha County (An et al. 2014; Wu et al. 2018). Further alignment where required was achieved using Simple Sequence Editor (SSE) Alignment software (Katoh et al. 2002). RDP4 (Martin et al. 2015) and GARD (Kosakovsky Pond et al. 2006) were used to test for recombination; in the RDP4 package, we analyzed the sequences with RDP, GENECONV, Bootscan, Maxchi, Chimera, SiScan and 3Seq, LARD, and Phylpro. Pairwise nucleotide p-distances were calculated using the program Compute Pairwise Distance within the MEGA v7.0 (Kumar et al. 2016) software package.

### 4.5 BEAST analysis

Temporal signaling of the gt6_Ref_ and gt6_Bai_ whole virus genome datasets was firstly verified using Phylostems (Doizy et al. 2020). We then used the Bayesian MCMC inference method, implemented in BEAST v1.10.4 (Drummond et al. 2012), to estimate the ancestral relationship of the sequenced gt6 genomes from the Baisha community. Three analyses were performed with the gt6_Ref_ and gt6_Bai_ whole virus genome datasets comprising a constant population size coalescent model and a Bayesian skyline coalescent model (10 groups) without partitioning, and a Bayesian skygrid model with partitioning. For each analysis, the SRD06 nucleotide partitioning model and an uncorrelated lognormal relaxed molecular clock were employed. A lognormal prior distribution (mean, 0.001; SD, 0.002) was used for the substitution rates. Throughout, convergence was assessed by examination of estimated effective sampling sizes (ESSs) and traces with 10–20 per cent burn-in using Tracer v1.7.1 (http://tree.bio.ed.ac.uk/software/tracer/). ESS values were predominantly ≥200 and at least 100 to ensure sufficient sampling. Two independent MCMC chains were run for 1 billion states for each of the two non-partitioned analyses and combined. Maximum clade credibility trees were calculated and annotated using TreeAnnotator 1.7.5 (http://tree.bio.ed.ac.uk/software/beast/) and visualized by FigTree v1.4.1 (http://tree.bio.ed.ac.uk/software/figtree/).

### 4.6 Site-specific selection in the HCV genome

We used PAL2NAL v14 (Suyama et al. 2006) to create a codon alignment based on a protein multiple sequence alignment which was translated from nucleotide to amino acid sequences by clcgenomics7 (https://www.qiagenbioinformatics.com/products/clc-genomics-workbench/). MEME (Detect Individual Sites Subject to Episodic Diversifying Selection) (Murrell et al. 2012), implemented in the Datamonkey package (http://www.datamonkey.org/), was used to detect adaptive evolution in the codon alignment. Sites with evidence of positive selection were then mapped to known epitopes taken from the HCV Immunology Database (hcv.lanl.gov and Yusim et al. (2005)).

### 4.7 Prevalence of RASs in HCV sequences

We investigated RAS considered to reduce susceptibility as published in EASL recommendations (https://easl.eu/wp-content/uploads/2018/10/HepC-English-report.pdf). The prevalence of RAS in the gt6_Bai_ sequences was determined for NS3, NS5A, and NS5B at a threshold of >15 per cent using the phdrSamReporter module within HCV-GLUE (Singer et al. 2019). The majority and minority variants for each sequence are reported relative to the amino acid position of the HCV gt1a strain H77 reference sequence. Accession numbers for sequences are shown in Supplementary Tables S1 and S3.

## Supplementary Material

veac012_Supp
