# Supplementary material for: The evolutionary dynamics and epidemiological history of hepatitis C virus genotype 6, including unique strains from the Li community of Hainan Island, China
Source: Virus Evol. 2022 Feb 16;8(1):veac012. doi: 10.1093/ve/veac012 (PMC9115904; doi:10.1093/ve/veac012)
Supplement: veac012_Supp [file veac012_supp.zip › Xu et al Supplementary Figs S1-7.pdf]

Supplementary Figure S1

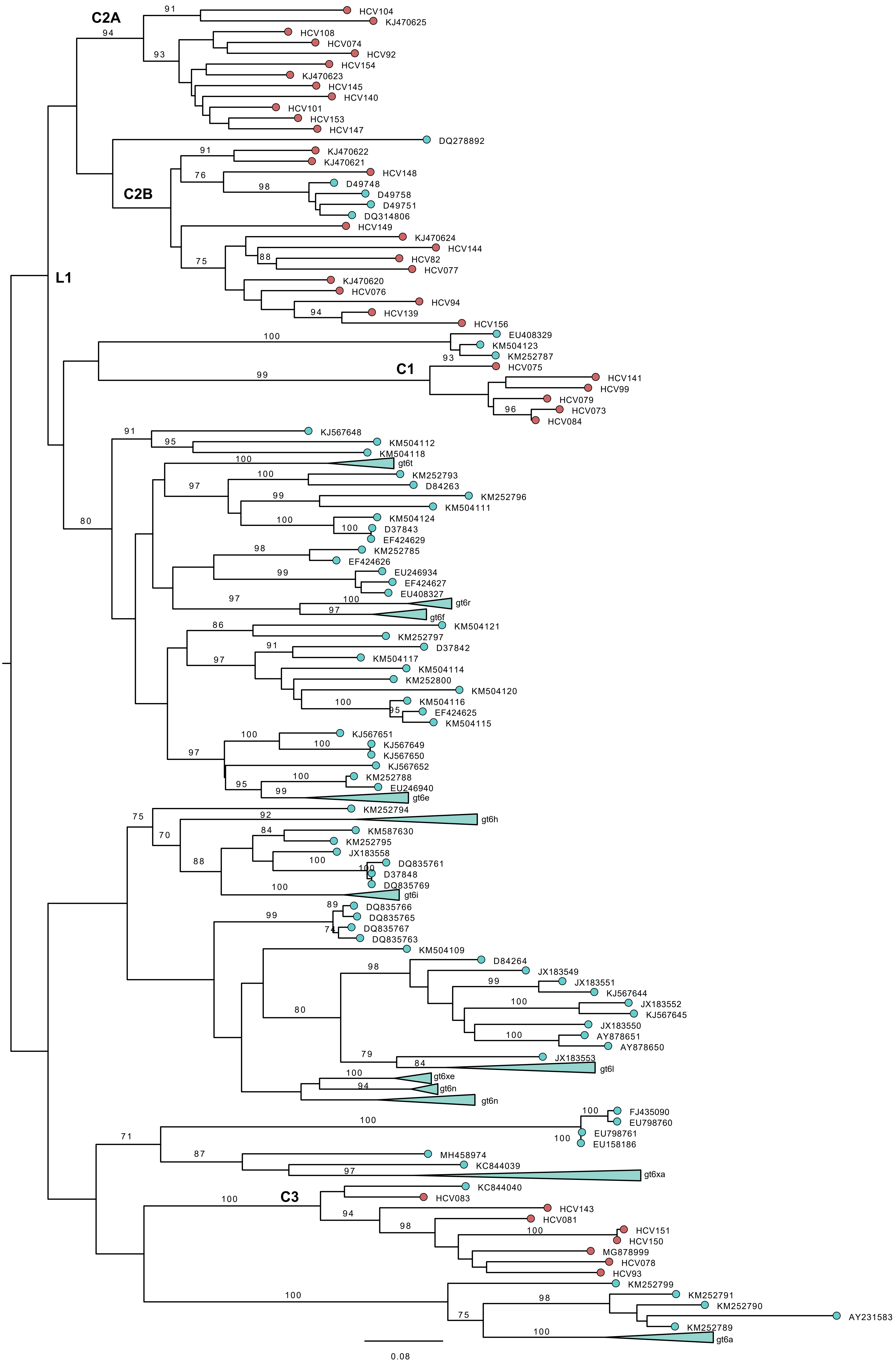

Supplementary Figure S2

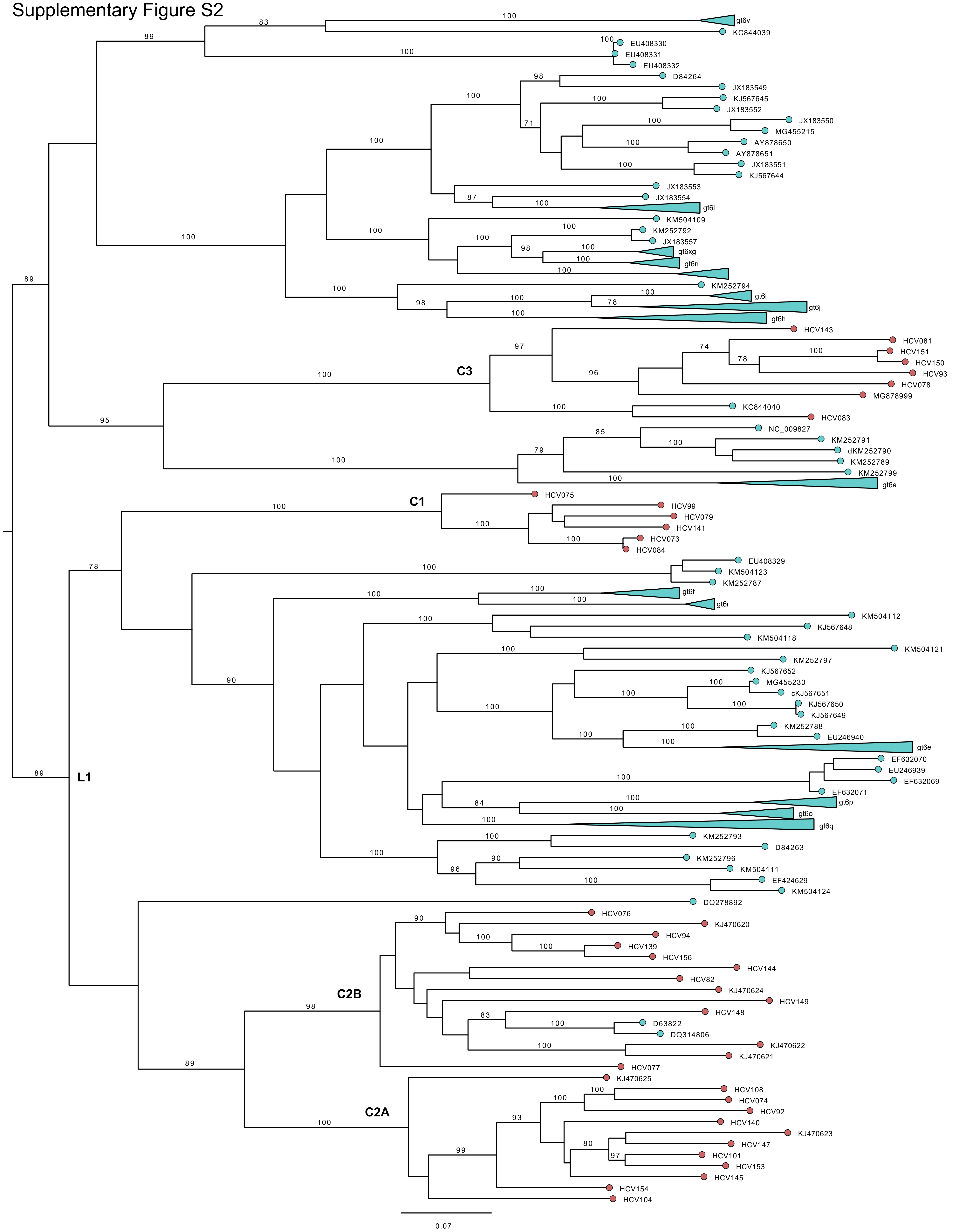

Supplementary Figure S3

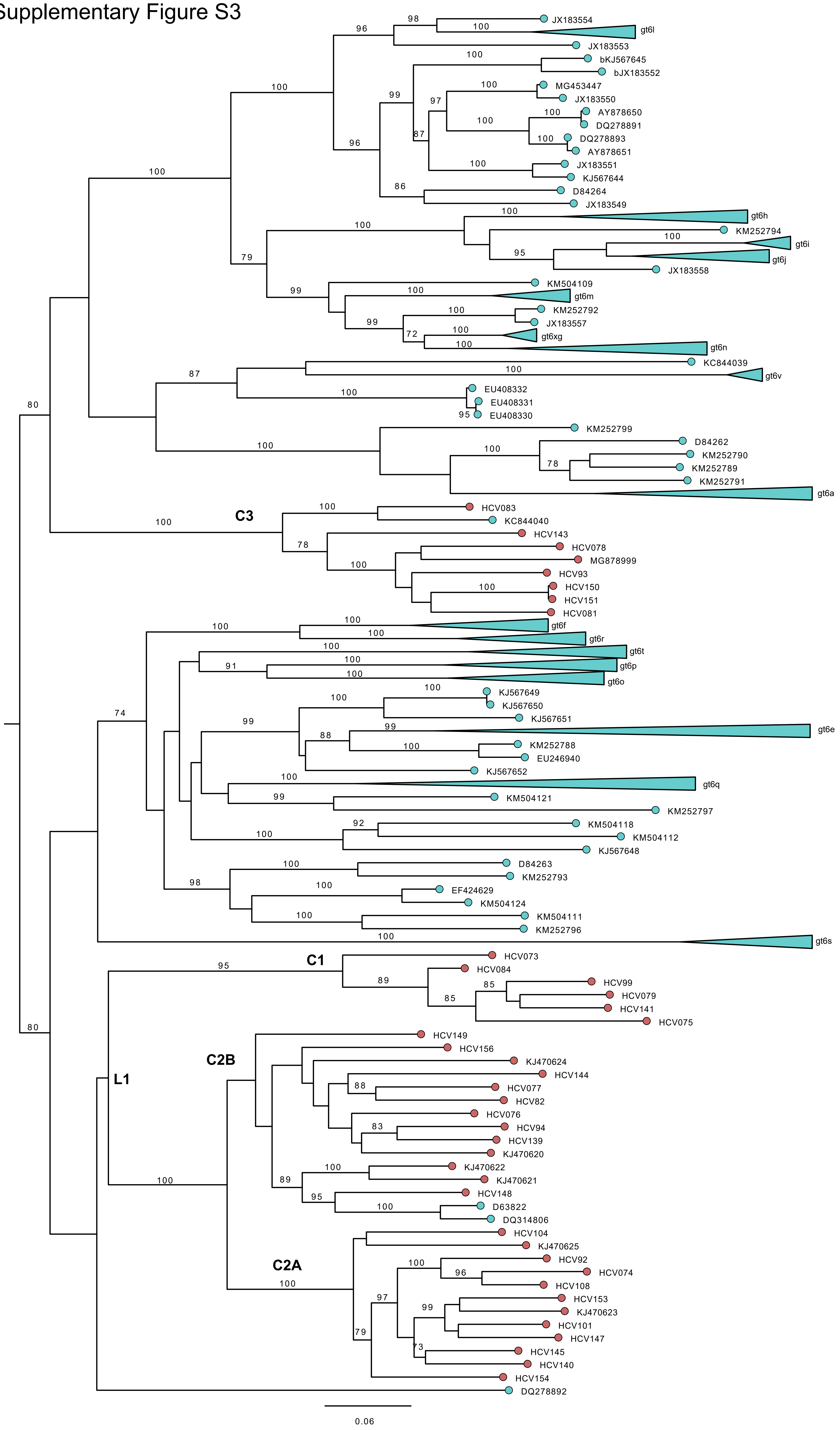

Supplementary Figure S4

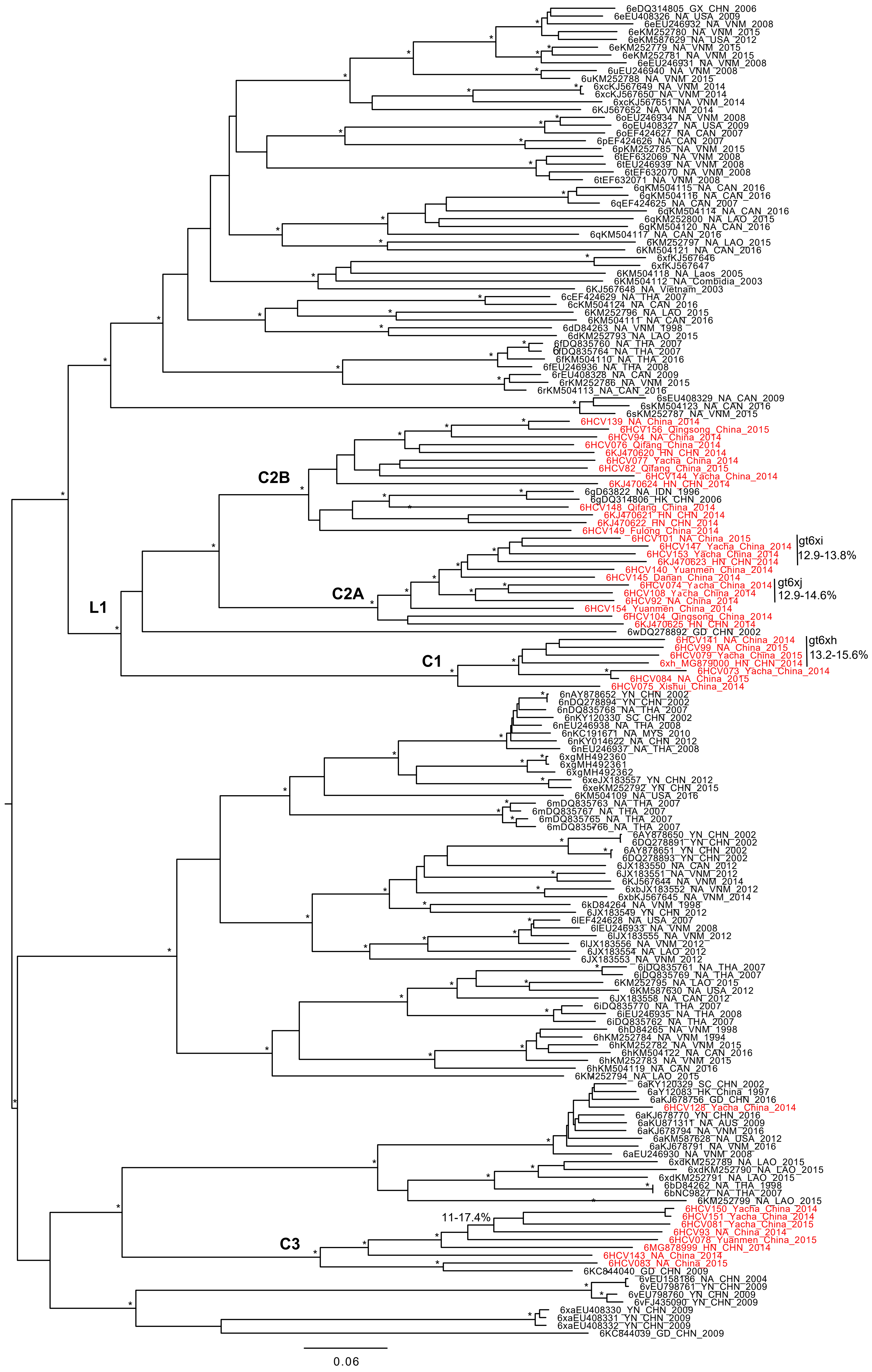

Supplementary Figure S5

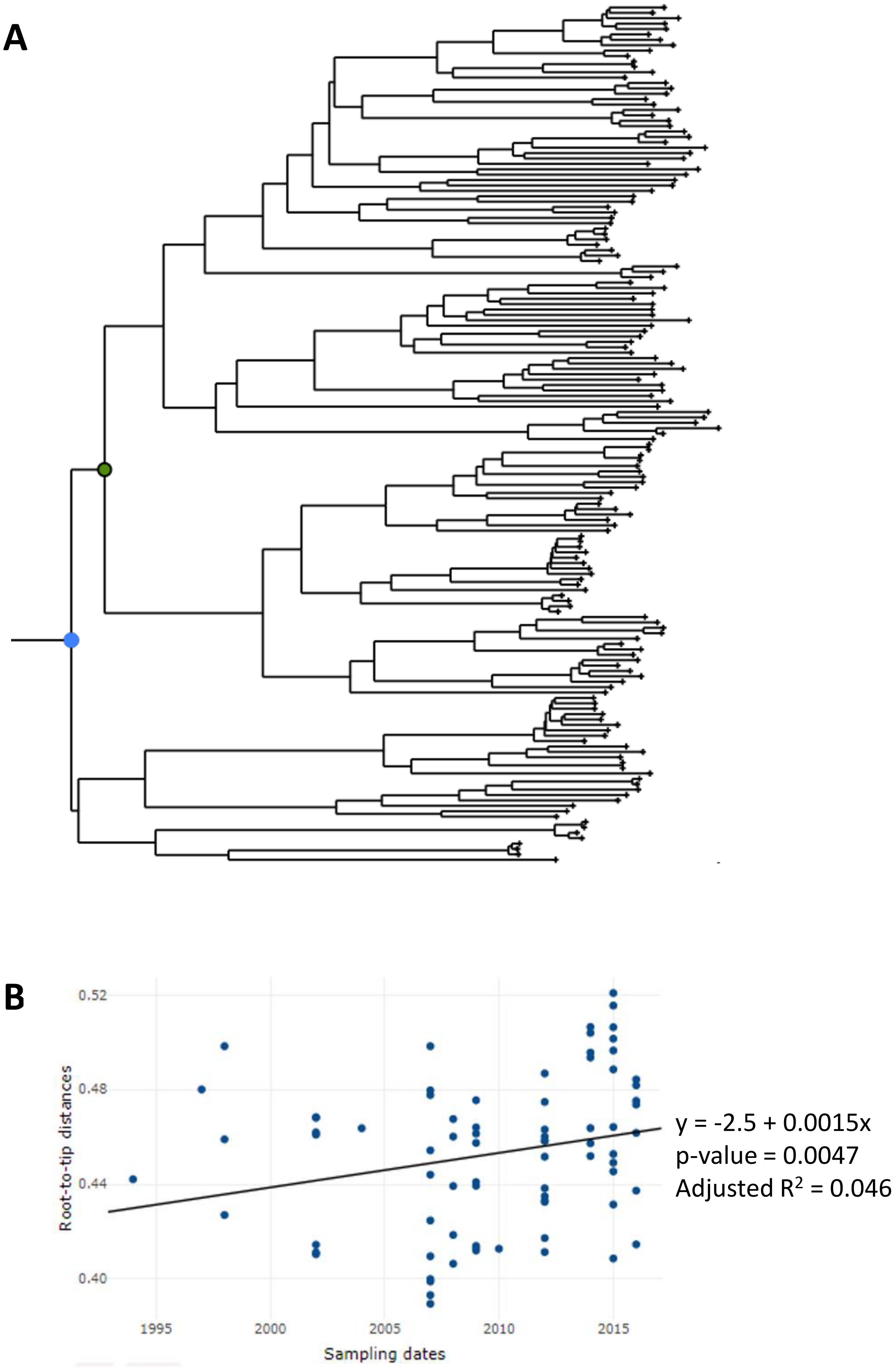

Supplementary Figure S6

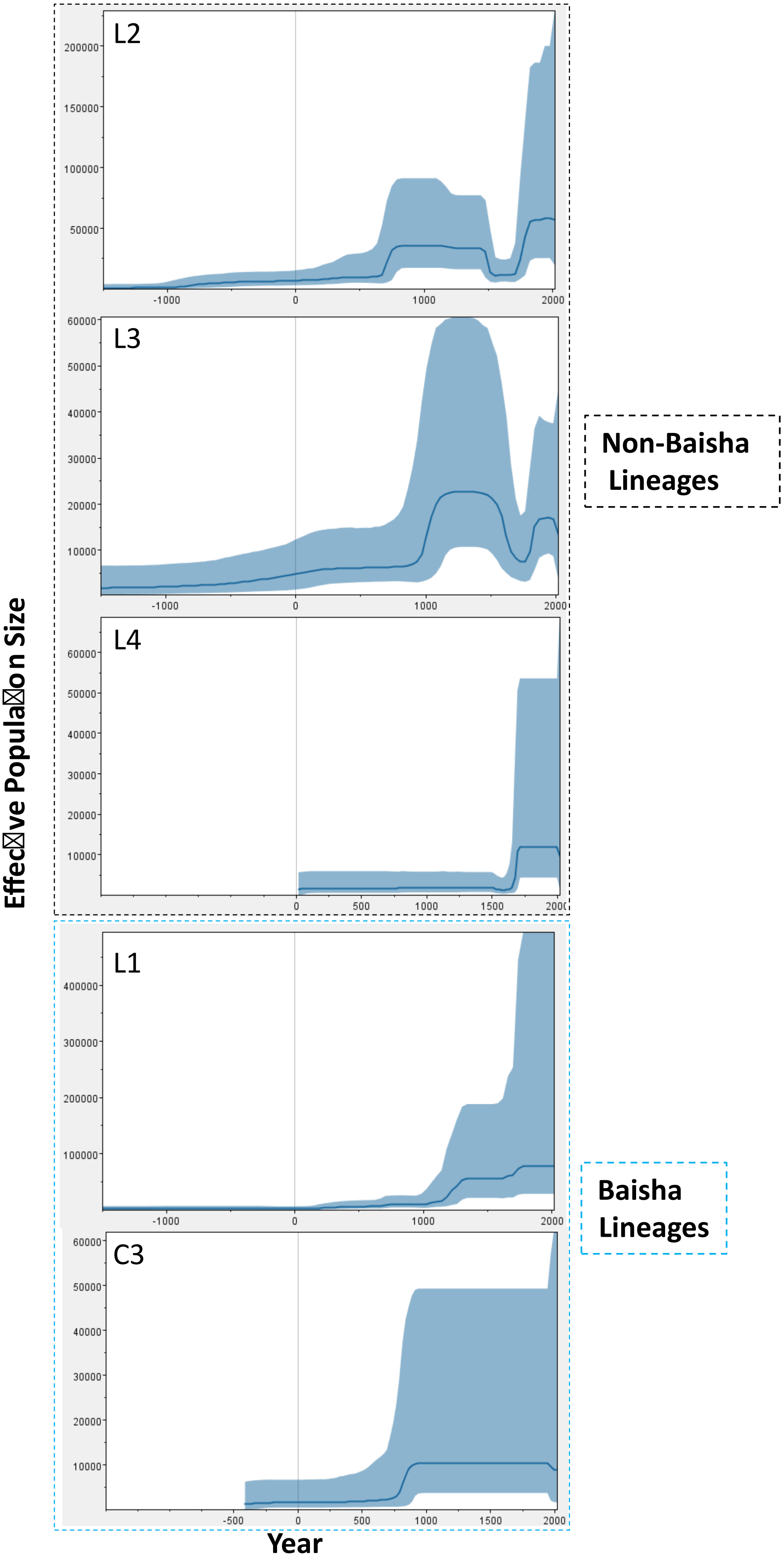

Supplementary Figure S7

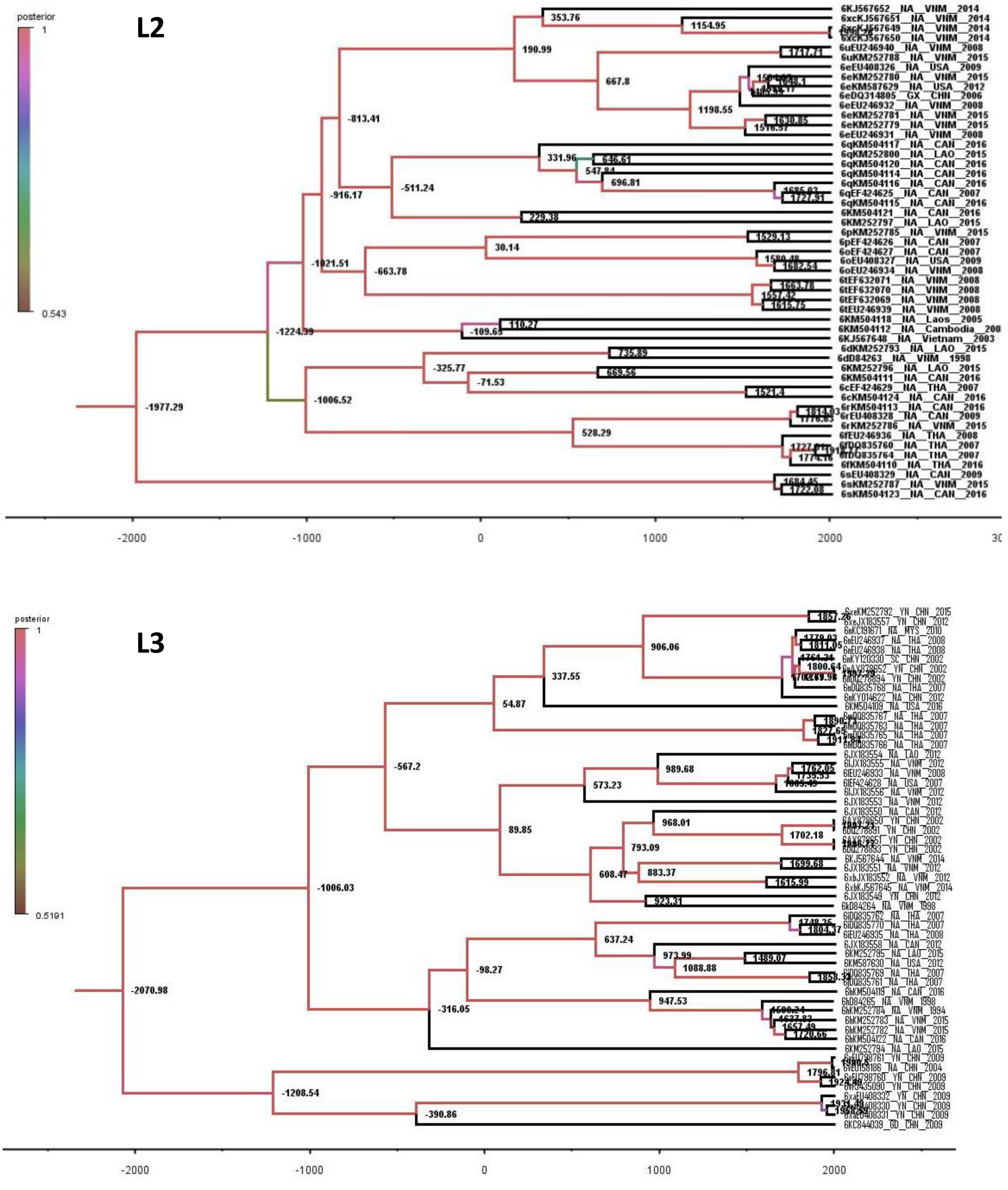

Year

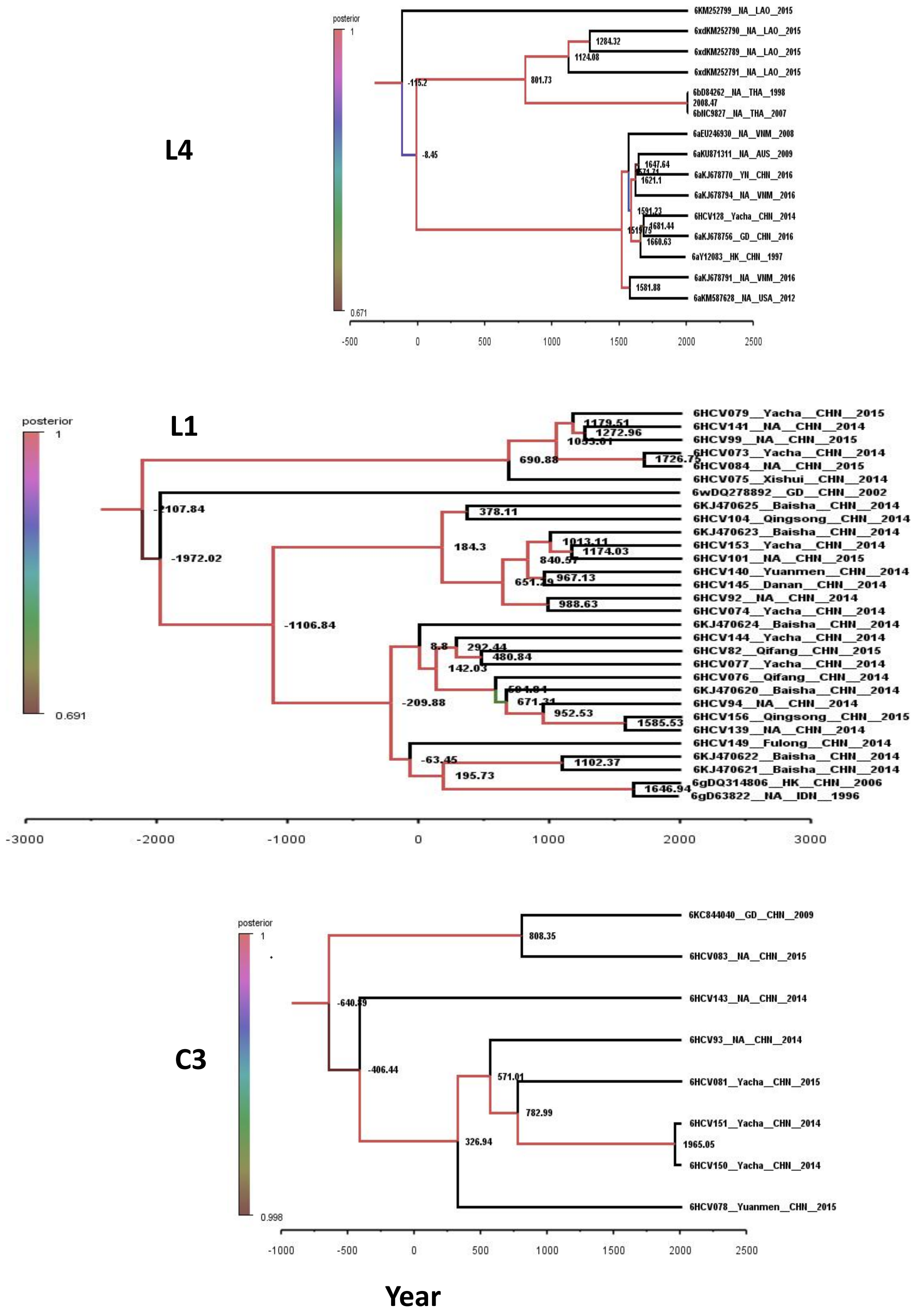

L4
posterior
1
0.671
6KM252799__NA__LAO__2015
6xdKM252790__NA__LAO__2015
6xdKM252789__NA__LAO__2015
6xdKM252791__NA__LAO__2015
6bD84262__NA__THA__1998
6bNC9827__NA__THA__2007
6aEU246930__NA__VNM__2008
6aKU871311__NA__AUS__2009
6aKJ678770__YN__CHN__2016
6aKJ678794__NA__VNM__2016
6HCV128__Yacha__CHN__2014
6aKJ678756__GD__CHN__2016
6aY12083__HK__CHN__1997
6aKJ678791__NA__VNM__2016
6aKM587628__NA__USA__2012
-500
0
500
1000
1500
2000
2500
L1
posterior
1
0.691
6HCV079__Yacha__CHN__2015
6HCV141__NA__CHN__2014
6HCV99__NA__CHN__2015
6HCV073__Yacha__CHN__2014
6HCV084__NA__CHN__2015
6HCV075__Xishui__CHN__2014
6wDQ278892__GD__CHN__2002
6KJ470625__Baisha__CHN__2014
6HCV104__Qingsong__CHN__2014
6KJ470623__Baisha__CHN__2014
6HCV153__Yacha__CHN__2014
6HCV101__NA__CHN__2015
6HCV140__Yuanmen__CHN__2014
6HCV145__Danan__CHN__2014
6HCV92__NA__CHN__2014
6HCV074__Yacha__CHN__2014
6KJ470624__Baisha__CHN__2014
6HCV144__Yacha__CHN__2014
6HCV82__Qifang__CHN__2015
6HCV077__Yacha__CHN__2014
6HCV076__Qifang__CHN__2014
6KJ470620__Baisha__CHN__2014
6HCV94__NA__CHN__2014
6HCV156__Qingsong__CHN__2015
6HCV139__NA__CHN__2014
6HCV149__Fulong__CHN__2014
6KJ470622__Baisha__CHN__2014
6KJ470621__Baisha__CHN__2014
6gDQ314806__HK__CHN__2006
6gD63822__NA__IDN__1996
-3000
-2000
-1000
0
1000
2000
3000
C3
posterior
1
0.998
6KC844040__GD__CHN__2009
6HCV083__NA__CHN__2015
6HCV143__NA__CHN__2014
6HCV93__NA__CHN__2014
6HCV081__Yacha__CHN__2015
6HCV151__Yacha__CHN__2014
6HCV150__Yacha__CHN__2014
6HCV078__Yuanmen__CHN__2015
-1000
-500
0
500
1000
1500
2000
2500
Year
