# Supplementary material for: The evolutionary dynamics and epidemiological history of hepatitis C virus genotype 6, including unique strains from the Li community of Hainan Island, China
Source: Virus Evol. 2022 Feb 16;8(1):veac012. doi: 10.1093/ve/veac012 (PMC9115904; doi:10.1093/ve/veac012)
Supplement: veac012_Supp [file veac012_supp.zip › Xu et al Supplementary Tables S1-4.docx]

**Supplementary Table S1. Age, gender and year of sample collection for patients in Baisha County from whom viral sequences were derived.**

| Sample IDs | Gender | Age | Collection  year | Accession number |
| --- | --- | --- | --- | --- |
| HCV073 | female | 77 | 2014 | MK328010.1 |
| HCV074 | female | 67 | 2014 | MK327985.1 |
| HCV075 | female | 83 | 2014 | MK327996.1 |
| HCV076 | female | 76 | 2014 | MK328005.1 |
| HCV077 | female | 85 | 2014 | MK327984.1 |
| HCV078 | male | 85 | 2015 | MK327993.1 |
| HCV079 | female | 61 | 2015 | MK328002.1 |
| HCV080 | female | 60 | 2015 | MK328020.1 |
| HCV081 | male | 49 | 2015 | MK328012.1 |
| HCV082 | female | 80 | 2015 | MK327988.1 |
| HCV083 | female | 79 | 2015 | MK328023.1 |
| HCV084 | NA | 79 | 2015 | MK328003.1 |
| HCV085 | male | 76 | 2015 | MK328024.1 |
| HCV086 | female | 82 | 2015 | MK327982.1 |
| HCV092 | male | 69 | 2014 | MK328009.1 |
| HCV093 | female | 89 | 2014 | MK328001.1 |
| HCV094 | male | 60 | 2014 | MK328019.1 |
| HCV096 | female | 90 | 2014 | MK327983.1 |
| HCV099 | male | 64 | 2015 | MK328006.1 |
| HCV101 | female | 66 | 2015 | MK327981.1 |
| HCV103 | female | 68 | 2014 | MK328008.1 |
| HCV104 | male | 77 | 2014 | MK327998.1 |
| HCV106 | male | 74 | 2014 | MK328017.1 |
| HCV108 | female | 82 | 2014 | MK327999.1 |
| HCV128 | NA | NA | 2014 | MK327986.1 |
| HCV138 | male | 77 | 2014 | MK327994.1 |
| HCV139 | female | 78 | 2014 | MK327992.1 |
| HCV140 | male | 70 | 2014 | MK328026.1 |
| HCV141 | female | 58 | 2014 | MK327989.1 |
| HCV142 | male | 87 | 2014 | MK328025.1 |
| HCV143 | female | 84 | 2014 | MK327991.1 |
| HCV144 | male | 83 | 2014 | MK328022.1 |
| HCV145 | male | 72 | 2014 | MK328014.1 |
| HCV146 | male | 86 | 2014 | MK328011.1 |
| HCV147 | female | 77 | 2014 | MK328007.1 |
| HCV148 | female | 84 | 2014 | MK328000.1 |
| HCV149 | male | 78 | 2014 | MK327997.1 |
| HCV150 | female | 77 | 2014 | MK327990.1 |
| HCV151 | female | 76 | 2014 | MK328021.1 |
| HCV152 | male | 76 | 2014 | MK327995.1 |
| HCV153 | male | 60 | 2014 | MK327987.1 |
| HCV154 | male | 94 | 2014 | MK328018.1 |
| HCV155 | male | 80 | 2014 | MK328016.1 |
| HCV156 | NA | 73 | 2015 | MK328013.1 |
| HCV157 | female | 84 | 2015 | MK328015.1 |
| HCV158 | male | 87 | 2015 | MK328004.1 |

**Supplementary Table S2. Consensus coverage data for HCV gt6_Bai_ sequences.**

| Sample ID^¶^ | Average depth | Min  depth | Max  depth | Coverage  (%) | Sequence length  (>9000bp) | Sequence Assembly | Gap location | Gap length (bp) |
| --- | --- | --- | --- | --- | --- | --- | --- | --- |
| HCV073 | 1572 | 5 | 5358 | 100.00 | Yes | De novo |  |  |
| HCV074 | 142 | 5 | 303 | 100.00 | Yes | De novo |  |  |
| HCV075 | 1367 | 11 | 4291 | 100.00 | Yes | De novo |  |  |
| HCV076 | 1307 | 8 | 4021 | 100.00 | Yes | De novo |  |  |
| HCV077 | 3449 | 5 | 11706 | 100.00 | Yes | De novo |  |  |
| HCV078 | 2054 | 5 | 5086 | 100.00 | Yes | De novo |  |  |
| HCV079 | 1340 | 7 | 3507 | 100.00 | Yes | De novo |  |  |
| HCV080 | 7172 | 0 | 14047 | 100.00 | No | De novo | E2/NS2/NS3/  NS4B/NS5A/  NS5B | 1153 |
| HCV081 | 538 | 10 | 1030 | 100.00 | Yes | De novo |  |  |
| HCV082 | 381 | 5 | 1323 | 100.00 | Yes | De novo |  |  |
| HCV083 | 1993 | 12 | 4874 | 100.00 | Yes | De novo |  |  |
| HCV084 | 3051 | 49 | 6207 | 100.00 | Yes | De novo |  |  |
| HCV085 | 14856 | 0 | 241101 | 94.89 | No | Reference | E1/E2/P7/NS2/  NS3 | 671 |
| HCV086 | 10625 | 0 | 237334 | 84.60 | No | Reference | E1/E2/P7/NS3/  NS5A | 334 |
| HCV092 | 220 | 5 | 4797 | 100.00 | Yes | De novo |  |  |
| HCV093 | 311 | 5 | 6822 | 100.00 | Yes | De novo |  |  |
| HCV094 | 2288 | 10 | 58865 | 100.00 | Yes | De novo |  |  |
| HCV096 | 450 | 0 | 10260 | 84.97 | No | De novo | E1/E2/P7/NS2/  NS3/NS5A | 884 |
| HCV099 | 5200 | 65 | 102686 | 100.00 | Yes | De novo |  |  |
| HCV101 | 64 | 0 | 40432 | 98.46 | Yes | De novo |  |  |
| HCV103 | 5507 | 0 | 86568 | 80.71 | No | Reference | E1/E2/P7/NS3/  NS4B | 1117 |
| HCV104 | 2544 | 8 | 66407 | 100.00 | Yes | De novo |  |  |
| HCV106 | 16 | 0 | 256 | 84.83 | No | Reference | E1/E2/P7/NS2/  NS4B/NS5A | 2990 |
| HCV108 | 2391 | 5 | 73271 | 100.00 | Yes | De novo |  |  |
| HCV128 | 5244 | 5 | 30922 | 100.00 | Yes | De novo |  |  |
| HCV138 | 8939 | 0 | 86623 | 54.67 | No | Reference | C/E1/E2/P7/NS2/NS3/NS4A/NS4B/  NS5A/NS5B | 916 |
| HCV139 | 19196 | 5 | 148042 | 100.00 | Yes | De novo |  |  |
| HCV140 | 22158 | 5 | 197089 | 100.00 | Yes | De novo |  |  |
| HCV141 | 146563 | 732 | 1252537 | 100.00 | Yes | De novo |  |  |
| HCV142 | 392 | 0 | 9273 | 62.66 | No | Reference | C/E2/P7/NS2/  NS4B/NS5A/  NS5B | 1618 |
| HCV143 | 28924 | 10 | 351858 | 100.00 | Yes | De novo |  |  |
| HCV144 | 2870 | 5 | 42891 | 100.00 | Yes | De novo |  |  |
| HCV145 | 12703 | 5 | 258908 | 98.37 | Yes | Reference |  |  |
| HCV146 | 318 | 0 | 4905 | 60.20 | No | Reference | E1/E2/P7/NS2/NS3/NS4A/NS4B/  NS5A/NS5B | 1088 |
| HCV147 | 10751 | 5 | 114882 | 100.00 | Yes | De novo |  |  |
| HCV148 | 14121 | 5 | 184505 | 94.88 | Yes | Reference |  |  |
| HCV149 | 17525 | 223 | 210040 | 100.00 | Yes | De novo |  |  |
| HCV150 | 11923 | 15 | 226506 | 100.00 | Yes | De novo |  |  |
| HCV151 | 6114 | 125 | 65104 | 99.99 | Yes | De novo |  |  |
| HCV152 | 3328 | 0 | 60022 | 81.43 | No | Reference | E1/E2/P7/NS2/  NS3/NS5A/NS5B | 621 |
| HCV153 | 19691 | 5 | 232480 | 100.00 | Yes | De novo |  |  |
| HCV154 | 7009 | 5 | 82632 | 95.55 | Yes | Reference |  |  |
| HCV155 | 310 | 0 | 5546 | 81.24 | No | Reference | E1/E2/P7/NS2/NS3/NS4A/NS4B/  NS5A/NS5B | 1495 |
| HCV156 | 2148 | 5 | 29168 | 100.00 | Yes | Reference |  |  |
| HCV157 | 3419 | 0 | 89396 | 80.29 | No | Reference | E1/E2/P7/NS2/  NS3/NS4A/NS5B | 405 |
| HCV158 | 864 | 0 | 10832 | 79.35 | No | Reference | E1/E2/P7/NS2/  NS3/NS5A | 825 |

^¶^ HCV gt6_Bai_ sequences containing gaps are indicated in grey and comprise the incomplete gt6 Baisha dataset.

**Supplementary Table S3. Details of HCV gt6 reference sequences (n=138) used for analysis.**

| Subtype | Accession number | Country | District/City/Town | Source of samples from non-Asian countries |
| --- | --- | --- | --- | --- |
| 6a | KJ678756^¶^ | CHN | GD |  |
| 6a | KJ678770 | CHN | YN |  |
| 6a* | KY120329 | CHN | SC |  |
| 6a | Y12083^¶^ | CHN | HK |  |
| 6a | KJ678791 | VNM | NA |  |
| 6a | KJ678794 | VNM | NA |  |
| 6a | EU246930^¶^ | VNM | NA |  |
| 6a | KM587628^¶^ | USA | NA | No details on country of origin |
| 6a | KU871311 | AUS | NA | No details on country of origin |
| 6b | D84262^¶^ | THA | NA |  |
| 6b | NC_009827 | THA | NA |  |
| 6c | EF424629^¶^ | THA | NA |  |
| 6c | KM504124 | CAN | NA | Individual from THA |
| 6d | D84263^¶^ | VNM | NA |  |
| 6d | KM252793 | LAO | NA |  |
| 6e | DQ314805^¶^ | CHN | GX |  |
| 6e | EU246931^¶^ | VNM | NA |  |
| 6e | EU246932^¶^ | VNM | NA |  |
| 6e | KM252779 | VNM | NA |  |
| 6e | KM252780 | VNM | NA |  |
| 6e | KM252781 | VNM | NA |  |
| 6e | EU408326 | USA | NA | Individual from Asia |
| 6e | KM587629 | USA | NA | No details on country of origin |
| 6f | DQ835760^¶^ | THA | NA |  |
| 6f | DQ835764 | THA | NA |  |
| 6f | EU246936^¶^ | THA | NA |  |
| 6f | KM504110 | THA | NA |  |
| 6g | D63822^¶^ | IDN | NA |  |
| 6g | DQ314806^¶^ | CHN | HK |  |
| 6h | D84265^¶^ | VNM | NA |  |
| 6h | KM252782 | VNM | NA |  |
| 6h | KM252783 | VNM | NA |  |
| 6h | KM252784 | VNM | NA |  |
| 6h | KM504119 | CAN | NA | Individual from LAO |
| 6h | KM504122 | CAN | NA | Individual from VNM |
| 6i | DQ835762^¶^ | THA | NA |  |
| 6i | DQ835770^¶^ | THA | NA |  |
| 6i | EU246935 | THA | NA |  |
| 6j | DQ835761^¶^ | THA | NA |  |
| 6j | DQ835769^¶^ | THA | NA |  |
| 6k | D84264^¶^ | VNM | NA |  |
| 6l | EU246933 | VNM | NA |  |
| 6l | JX183555 | VNM | NA |  |
| 6l | JX183556^¶^ | VNM | NA |  |
| 6l | EF424628^¶^ | USA | NA | Individual from Asia |
| 6m | DQ835763 | THA | NA |  |
| 6m | DQ835765 | THA | NA |  |
| 6m | DQ835766^¶^ | THA | NA |  |
| 6m | DQ835767^¶^ | THA | NA |  |
| 6n | AY878652 | CHN | YN |  |
| 6n | DQ278894^¶^ | CHN | YN |  |
| 6n | KY014622 | CHN | NA |  |
| 6n | KY120330 | CHN | SC |  |
| 6n | DQ835768^¶^ | THA | NA |  |
| 6n | EU246937 | THA | NA |  |
| 6n | EU246938^¶^ | THA | NA |  |
| 6n | KC191671 | MYS | NA |  |
| 6o | EU246934^¶^ | VNM | NA |  |
| 6o | EF424627^¶^ | CAN | NA | Caucasian individual |
| 6o | EU408327 | USA | NA | Individual from Asia |
| 6p | KM252785 | VNM | NA |  |
| 6p | EF424626^¶^ | CAN | NA | Individual from VNM |
| 6q | KM252800 | LAO | NA |  |
| 6q | EF424625^¶^ | CAN | NA | Individual from KH |
| 6q | KM504114 | CAN | NA | Individual from KH |
| 6q | KM504115 | CAN | NA | Individual from KH |
| 6q | KM504116 | CAN | NA | Individual from KH |
| 6q | KM504117 | CAN | NA | Individual from KH |
| 6q | KM504120 | CAN | NA | Individual from VNM |
| 6r | KM252786 | VNM | NA |  |
| 6r | EU408328^¶^ | CAN | NA | Individual from Asia |
| 6r | KM504113 | CAN | NA | Individual from KH |
| 6s | KM252787 | VNM | NA |  |
| 6s | EU408329^¶^ | CAN | NA | Individual from Asia |
| 6s | KM504123 | CAN | NA | Individual from KH |
| 6t | EF632069 | VNM | NA |  |
| 6t | EF632070 | VNM | NA |  |
| 6t | EF632071^¶^ | VNM | NA |  |
| 6t | EU246939^¶^ | VNM | NA |  |
| 6u | EU246940^¶^ | VNM | NA |  |
| 6u | KM252788 | VNM | NA |  |
| 6v | EU158186^¶^ | CHN | NA |  |
| 6v | EU798760^¶^ | CHN | YN |  |
| 6v | EU798761^¶^ | CHN | YN |  |
| 6v | FJ435090 | CHN | YN |  |
| 6w | DQ278892^¶^ | CHN | GD |  |
| 6xa | EU408330^¶^ | CHN | YN |  |
| 6xa | EU408331^¶^ | CHN | YN |  |
| 6xa | EU408332^¶^ | CHN | YN |  |
| 6xb* | JX183552^¶^ | VNM | NA |  |
| 6xb | KJ567645^¶^ | VNM | NA |  |
| 6xc | KJ567649^¶^ | VNM | NA |  |
| 6xc | KJ567650^¶^ | VNM | NA |  |
| 6xc | KJ567651^¶^ | VNM | NA |  |
| 6xd | KM252789^¶^ | LAO | NA |  |
| 6xd | KM252790^¶^ | LAO | NA |  |
| 6xd | KM252791^¶^ | LAO | NA |  |
| 6xe | JX183557^¶^ | CHN | YN |  |
| 6xe | KM252792^¶^ | CHN | YN |  |
| 6xf* | KJ567646^¶^ | VNM | NA |  |
| 6xf* | KJ567647^¶^ | VNM | NA |  |
| 6xg* | MH492360^¶^ | MYS | NA |  |
| 6xg* | MH492361^¶^ | MYS | NA |  |
| 6xg* | MH492362^¶^ | MYS | NA |  |
| 6xh* | MG879000^¶^ | CHN | HN |  |
| 6 | KJ470620^¶^ | CHN | HN |  |
| 6 | KJ470621^¶^ | CHN | HN |  |
| 6 | KJ470622^¶^ | CHN | HN |  |
| 6 | KJ470623^¶^ | CHN | HN |  |
| 6 | KJ470624^¶^ | CHN | HN |  |
| 6 | KJ470625^¶^ | CHN | HN |  |
| 6* | MG878999^¶^ | CHN | HN |  |
| 6 | KC844039^¶^ | CHN | GD |  |
| 6 | KC844040^¶^ | CHN | GD |  |
| 6 | AY878650 | CHN | YN |  |
| 6 | AY878651 | CHN | YN |  |
| 6 | DQ278891^¶^ | CHN | YN |  |
| 6 | DQ278893^¶^ | CHN | YN |  |
| 6 | JX183549^¶^ | CHN | YN |  |
| 6 | KJ567644^¶^ | VNM | NA |  |
| 6 | KJ567652^¶^ | VNM | NA |  |
| 6 | JX183551^¶^ | VNM | NA |  |
| 6 | JX183553^¶^ | VNM | NA |  |
| 6 | KC567648 | VNM | NA |  |
| 6 | JX183554^¶^ | LAO | NA |  |
| 6 | KM252794^¶^ | LAO | NA |  |
| 6 | KM252795^¶^ | LAO | NA |  |
| 6 | KM252796 | LAO | NA |  |
| 6 | KM252797 | LAO | NA |  |
| 6 | KM252799 | LAO | NA |  |
| 6 | KM504118 | LAO | NA |  |
| 6 | JX183550^¶^ | CAN | NA | Individual from KH |
| 6 | JX183558^¶^ | CAN | NA | Individual from THA |
| 6 | KM504111 | CAN | NA | Individual from KH |
| 6 | KM504121^¶^ | CAN | NA | Individual from LAO |
| 6 | KM504112 | CAN | NA | Individual from KH |
| 6 | KM504109 | USA | NA | Individual from MYM |
| 6 | KM587630 | USA | NA | No details on country of origin |

Reference sequences collected from China, including those from Hainan Island (HN), are highlighted in grey. For sequences reported from non-Asian countries (AUS, CAN and USA), the country of origin for individuals who provided samples is shown in the final column.

^¶^ Sequences included for the ML tree in Fig 2A.

* Excluded from MCMC analyses due to sequence gaps.

CHN: China; AUS: Australia; CAN: Canada; IDN: Indonesia; LAO: Lao; MYS: Malaysia; MYM; Myanmar; THA: Thailand; USA: United States; VNM: Vietnam; KH: Cambodia; GD: Guangdong; GX: Guangxi; HK: Hong Kong; HN: Hainan Island; SC: Sichuan; SZ: Shenzhen; YN: Yunnan.

**Supplementary Table S4. Substitution Rate, Coefficient of Variation and Covariance of HCV gt6 genome regions.**

| Region | Substitution Rate (s/s/y)* [HPD^95^] | Coefficient of Variation [HPD^95^] | Covariance [HPD^95^] |
| --- | --- | --- | --- |
| Coding Region | 1.20 x 10^-4^  [1.00 x 10^-4^ – 1.65 x 10^-4^] | 0.236  [0.204 – 0.27] | -5.70 x 10^-2^  [-0.17 – 0.05] |
| 5’UTR | 1.02 x 10^-5^  [7.12 x 10^-6^ – 1.37 x 10^-5^] | 2.008  [1.309 – 2.844] | 2.11 x 10^-2^  [-0.07 – 0.13] |
| Core | 8.90 x 10^-5^  [7.81 x 10^-5^ – 1.00 x 10^-4^] | 0.526  [0.437 – 0.616] | -2.97 x 10^-2^  [-0.13 – 0.07] |
| E1 | 1.84 x 10^-4^  [1.69 x 10^-4^ – 1.98 x 10^-4^] | 0.3  [0.255 – 0.350] | 5.17 x 10^-3^  [-0.09 – 0.11] |
| E2 | 3.07 x 10^-4^  [2.79 x 10^-4^ – 3.34 x 10^-4^] | 0.379  [0.338 – 0.419] | 6.86 x 10^-2^  [-0.03 – 0.17] |
| P7 | 2.10 x 10^-4^  [1.87 x 10^-4^ – 2.41 x 10^-4^] | 0.285  [0.207 – 0.378] | 2.61 x 10^-2^  [-0.09 – 0.13] |
| NS2 | 1.88 x 10^-4^  [1.72 x 10^-4^ – 2.02 x 10^-4^] | 0.235  [0.188 – 0.279] | -3.85 x 10^-2^  [-0.15 – 0.06] |
| NS3 | 1.61 x 10^-4^  [1.53 x 10^-4^ – 1.69 x 10^-4^]# | 0.217  [0.184 – 0.251] | -1.74 x 10^-3^  [-0.10 – 0.11] |
| NS4A | 1.56 x 10^-4^  [1.35 x 10^-4^ – 1.74 x 10^-4^] | 0.303  [0.184 – 0.427] | -6.62 x 10^-3^  [-0.12 – 0.10] |
| NS4B | 1.40 x 10^-4^  [1.32 x 10^-4^ – 1.49 x 10^-4^]# | 0.216  [0.171 – 0.266] | -5.10 x 10^-3^  [-0.11 – 0.09] |
| NS5A | 1.66 x 10^-4^  [1.57 x 10^-4^ – 1.75 x 10^-4^] | 0.225  [0.190 – 0.261] | -2.28 x 10^-2^  [-0.12 – 0.08] |
| NS5B | 1.38 x 10^-4^  [1.29 x 10^-4^ – 1.48 x 10^-4^]# | 0.654  [0.578 – 0.720] | -1.82 x 10^-2^  [-0.10 – 0.08] |

* Substitutions/site/year

#ESS was 124 – 183
